# Cooperative Retraction of Bundled Type IV Pili Enables Nanonewton Force Generation

**DOI:** 10.1371/journal.pbio.0060087

**Published:** 2008-04-15

**Authors:** Nicolas Biais, Benoît Ladoux, Dustin Higashi, Magdalene So, Michael Sheetz

**Affiliations:** 1 Department of Biological Sciences, Columbia University, New York, New York, United States of America; 2 Matière et Systèmes Complexes, CNRS UMR7057/Université Paris 7, Bâtiment Condorcet, 75205 Paris cedex 13, France; 3 Department of Immunobiology, BIO5 Institute, University of Arizona, Tucson, Arizona, United States of America; Adolf-Butenandt-Institut Zellbiologie, Germany

## Abstract

The causative agent of gonorrhea, Neisseria gonorrhoeae, bears retractable filamentous appendages called type IV pili (Tfp). Tfp are used by many pathogenic and nonpathogenic bacteria to carry out a number of vital functions, including DNA uptake, twitching motility (crawling over surfaces), and attachment to host cells. In N. gonorrhoeae, Tfp binding to epithelial cells and the mechanical forces associated with this binding stimulate signaling cascades and gene expression that enhance infection. Retraction of a single Tfp filament generates forces of 50–100 piconewtons, but nothing is known, thus far, on the retraction force ability of multiple Tfp filaments, even though each bacterium expresses multiple Tfp and multiple bacteria interact during infection. We designed a micropillar assay system to measure Tfp retraction forces. This system consists of an array of force sensors made of elastic pillars that allow quantification of retraction forces from adherent N. gonorrhoeae bacteria. Electron microscopy and fluorescence microscopy were used in combination with this novel assay to assess the structures of Tfp. We show that Tfp can form bundles, which contain up to 8–10 Tfp filaments, that act as coordinated retractable units with forces up to 10 times greater than single filament retraction forces. Furthermore, single filament retraction forces are transient, whereas bundled filaments produce retraction forces that can be sustained. Alterations of noncovalent protein–protein interactions between Tfp can inhibit both bundle formation and high-amplitude retraction forces. Retraction forces build over time through the recruitment and bundling of multiple Tfp that pull cooperatively to generate forces in the nanonewton range. We propose that Tfp retraction can be synchronized through bundling, that Tfp bundle retraction can generate forces in the nanonewton range in vivo, and that such high forces could affect infection.

## Author Summary

Type IV pili are filamentous appendages borne by a large number of pathogenic and nonpathogenic bacteria. They play crucial roles in basic microbial processes such as surface motility, virulence, and DNA exchange. Neisseria gonorrhoeae, the causative agent of gonorrhea, can extend and retract these long, thin threads—around 6 nm in diameter and up to 30 μm long—to explore and pull on the environment. The retraction of one N. gonorrhoeae pilus filament can exert forces of 50–100 piconewtons, or roughly 10,000 times the bacterium's bodyweight. The bacteria can exert those forces on human cells that they infect, and force has been shown to be an important parameter in their infectivity. We use a micropillar assay system to show that N. gonorrhoeae cells can exert even higher forces by forming bundles of 8–10 filaments that act as coordinated retractable units. The bacteria can thus achieve forces in the nanonewton range (or 100,000 times their bodyweight) making them the strongest microscale elements known to date. This study demonstrates the power and cooperativity of pilus nanomotors and opens new territories for the exploration of force-mediated bacteria–host-cell interactions.

## Abstract 3

To make its way into cells,*Neisseria gonorrhoeae*, the pathogen that causes gonorrhea, may exert nanonewton forces through cooperative bundling of pilus fibers.

## Introduction

Type IV pili (Tfp) are bacterial appendages with important biological functions, including motility, DNA transformation, and virulence [1]. Among the most-studied Tfp are those elaborated by N. gonorrhoeae, which display Tfp over its entire surface. The N. gonorrhoeae Tfp is a helical polymer of pilin [2] subunits ∼6 nm wide and several micrometers long [3]. Tfp enable N. gonorrhoeae, as well as other bacteria, to attach to and pull on host cells or other substrates [4–6]. Cycles of Tfp extension, substrate binding, and retraction enable N. gonorrhoeae to crawl (twitching motility) [7,8]. Interest in the role of Tfp in infection was renewed by recent studies linking Tfp retraction force to the induction of epithelial cell responses [9,10]. The motor protein PilT [11,12] is essential for pilus retraction, and measurements using optical tweezers indicate that retraction of a single Tfp filament by a single PilT motor generates forces of 50–100 piconewtons (pN) [13]. These assays were limited to measuring retraction forces from individual diplococci. During infection of human tissue culture cells, N. gonorrhoeae aggregate into microcolonies via retracting Tfp [14,15]. Because the retraction forces exerted by the bacteria have been shown to play a key role in their interaction with host cells [9,10], it is important to determine the range of forces that can be exerted by bacteria under infection conditions. Given the force limitation of optical tweezers, which cannot measure forces above 200 pN, and the potential of multiple Tfp contributing to retraction forces, we developed a new methodology to measure high retraction forces. Here, we demonstrate the ability of N. gonorrhoeae to impose tremendous forces (nanonewton [nN] range) on its substrate and allow us a more precise and comprehensive view of the physical role of Tfp in N. gonorrhoeae pathogenesis.

## Results and Discussion

### Measurement of Forces Exerted by Tfp

**Figure 1:**
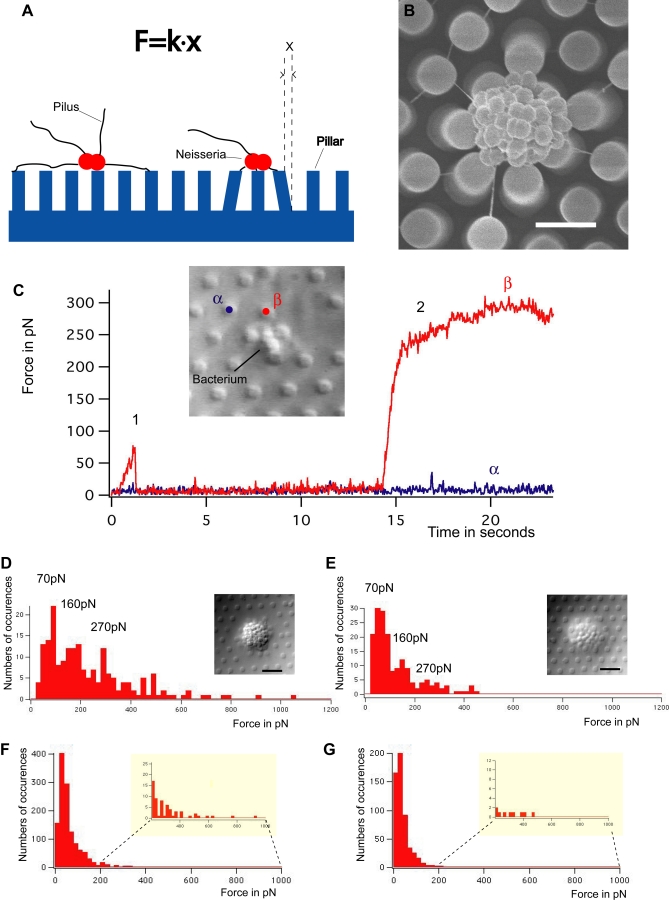
N. gonorrhoeae Tfp Can Exert Forces in the nN Range (A) Schematic of the force measurement assay used in this study. (B) SEM micrograph of a microcolony with Tfp pulling on pillars (here the pillars are molded in polydimethylsiloxane, a widely used, silicon-based polymer, to enable SEM). Scale bar = 2 μm. (C) Time course of the force exerted on a pillar. (1, trace β): short-lived, low-force pulling event. (2, trace β): long-lived, high-force event. Trace α shows a neighboring pillar that did not move. (D and E), Histograms of the forces recorded in static studies for WT MS11 in DMEM (D) or in DMEM + BSA (E). Inserts are differential interference contrast images of representative microlonies in the samples. Scale bar=5 μm. (F and G), Histograms of the forces recorded in dynamic studies of WT MS11 in DMEM (F) or DMEM + BSA (G). Inserts represent close-ups of the tail of each force distribution curve (forces between 200 pN and 1000 pN).

To measure high Tfp retraction forces from N. gonorrhoeae, we developed a new assay based on elastic micropillars by modifying previously published methods [16,17]. Our force sensor consists of an array of evenly spaced micropillars made of an elastic hydrogel (Figure 1A and 1B, and Video S1, to see a video of the device). A pulling force exerted on a pillar, such as that imposed by an attached pilus fiber, causes a displacement of the pillar tip. This displacement can be correlated to the force applied on the pillar after proper calibrations. Modifying the elastic properties of the gel, we were able to vary the stiffness of the pillars from 100 to 500 pN/μm. This allowed us to record forces in the pN to nanonewton (nN) range. Bacteria were seeded on these force sensors in Dulbecco's modified Eagle's medium (DMEM) tissue culture medium, allowed to interact with each other as in an infection assay, and the displacement of the pillars adjacent to the bacteria was recorded. The motion of the pillars revealed two types of pulling behavior from the bacteria. As reported previously [13], we observed transient retraction events—or pulls—lasting anywhere from a few tenths of a second to several seconds, with a magnitude <100 pN (1, trace β in Figure 1C). We also recorded much longer retraction events lasting from a few seconds to several hours, which exerted much higher forces of between 200 pN and 1 nN (2, trace β in Figure 1C). Until now, retraction events of this magnitude have never been seen in N. gonorrhoeae. In all cases, only a few pillars were observed to be moving at a given time relative to stationary neighboring pillars (trace α in Figure 1C), showing that these retraction events were specific to particular pillars. Tfp retraction forces under those conditions were up to 10 times higher than those recorded for a single Tfp filament.

We next asked if there was a regular pattern associated with these high-amplitude forces. We used two methods for recording the forces exerted on a pillar (see Figure S1 for details). We used either video rate (30 Hz) imaging to capture the motion of a pillar's tip (dynamic studies), or recording the deflection of the pillars by taking a picture at the base and at the tip of the pillar in fixed samples (static studies). Dynamic studies allowed us to monitor accurately short-lived pulling events, whereas the static studies allowed us to view long-term pulling events. Histograms of both types of recordings revealed a low force peak that was characteristic of single Tfp retraction events (70 ± 20 pN for static studies, and 40 ± 20 pN for dynamic studies) (Figure 1D and 1F, respectively). The two types of force recordings differed in the percentage of higher force measurements as, for instance, the maximum force (∼1 nN) events represent ∼1% of the measurements in the static studies and only 0.1% in the dynamical studies (Figure 1D and 1F, respectively). In the force histograms from the fixed samples, we observed a number of peaks with values that were roughly multiples of a single filament retraction force (Figure 1D). These peaks indicated that higher force retractions may involve the simultaneous pulling of 2, 3, 4 or more Tfp. A careful examination of rare dynamic events lends additional support to this possibility (Figure S2). In most cases, force increased in a step-wise fashion, commonly at increments of 70–100 pN.

Why have such high forces (nN) not been recorded before? One possible explanation is that previous assays were conducted in medium containing a high concentration of bovine serum albumin (BSA; 1 mg/ml) [7,13]. In many cases, BSA has the potential to prevent nonspecific interactions between proteins, and this could apply to proteinaceous assemblies such as the Tfp. To test the effect of BSA on Tfp retraction, we conducted our measurements in the presence of BSA. Under those conditions, we obtained very different histograms (Figure 1E). We observed a primary peak in the histogram that was much higher than the primary peak recorded in the absence of BSA, and we recorded subsequent peaks that were much smaller than those measured in the absence of BSA. Thus, low-force retraction events were more numerous in the presence of BSA. Furthermore, the maximum forces measured in the presence of BSA never reached the levels observed in the medium without BSA either in static or dynamic measurements (Figure 1F and 1G). Thus, the addition of BSA to the assay medium inhibits bacteria from pulling with high forces.

### Structure of the Pulling Tfp

**Figure 2:**
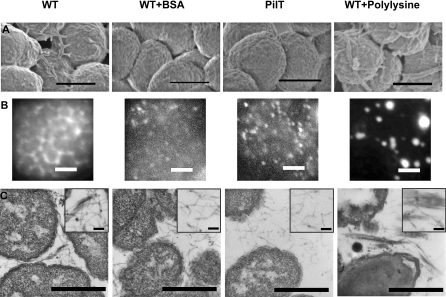
Tfp from WT MS11 and MS11PilT in Different States of Bundling WT: MS11 not exposed to BSA; WT + BSA: MS11 incubated with BSA; PilT: MS11PilT not exposed to BSA; WT + polylysine: MS11 incubated with polylysine. (A) SEM micrographs. Scale bar = 500 nm. (B) Fluorescent images of microcolonies immunostained with monoclonal anti-Tfp antibody (anti-SM1). Scale bar = 2 μm. (C) Thin-section EM image of Tfp and Tfp bundles. Insert in each panel represents a higher magnification image of representative Tfp in the sample. Note the bundles in the WT and WT+polylysine samples that are not present in the WT+BSA and pilT samples. Scale bar = 500nm. Scale bar = 50 nm in the inserts.

Because of the possibility that Tfp could break as well as bundle [18–20], we used thin section and scanning electron microscopy (SEM) to reveal differences in the Tfp structure of N. gonorrhoeae microcolonies incubated in DMEM with or without BSA. Single Tfp filaments were present on bacteria incubated with and without BSA, as judged by thin-section EM. However, in the absence of BSA, Tfp bundled along their long axis to form rope-like structures (Figure 2A and 2C). Using SEM, bundles were seen emanating from single diplococci as well as from junctions between different cells. Single Tfp were not apparent in the SEM studies. Thus, Tfp bundling can be prevented by the addition of BSA to the culture medium. These differences in bundling phenotypes were also observed in microcolonies immunostained with a monoclonal antibody against Tfp (Figure 2B). In untreated medium (no BSA), microcolony Tfp staining gave an intense fluorescence at the interstices of the bacteria that was suggestive of bundles. In contrast, Tfp fluorescence in BSA-treated medium yielded microcolony Tfp staining that was diffuse (Figure 2B). Interestingly, Tfp retraction was apparently necessary for bundle formation, since Tfp from PilT-null mutant microcolonies had a similar appearance to wild-type (WT) microcolonies assayed in BSA-containing medium (Figure 2A–2C). We have thus established a direct correlation between Tfp bundling and the generation of high retraction forces. The occurrence of bundled Tfp have been reported previously in N. gonorrhoeae [20] and N. meningitidis [21].

**Figure 3:**
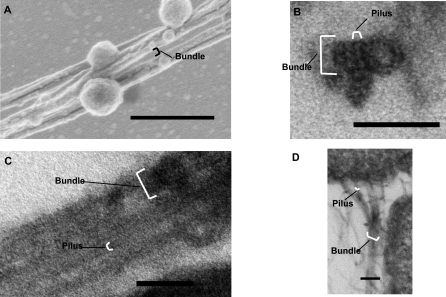
Structure of Tfp Bundles (A) SEM picture of a collection of bundles in a polylysine-treated sample. Scale bar = 500 nm. (B) Thin-section microscopy image of the cross-section of a collection of bundles in a polylysine-treated sample (here composed of two bundles). Scale bar = 50 nm. (C) Thin-section microscopy image of the long axis of a collection of bundles in a polylysine-treated sample (here composed of three bundles). Scale bar = 50 nm. (D) Thin-section microscopy image of a bundle attached to a bacterium in a sample incubated with DMEM only. Scale bar = 50 nm.

To understand further the process of Tfp bundle formation, we promoted bundling by adding soluble polylysine (30 μg/ml) to the incubation medium. Polylysine is a polymer that bears multiple positive charges that can be used to promote the binding between negatively charged entities. This treatment yielded thicker, ropelike Tfp structures (Figure 2A). The microcolonies were smaller and the bacteria within them were less tightly packed, compared with untreated samples (Biais and Sheetz, unpublished data). After 3 h of incubation, we noted an almost complete cessation of retraction events in the polylysine-treated samples (Video S4), compared with WT (Video S2) and WT+BSA (Video S3) samples. Thus, the larger Tfp aggregates that were formed in the presence of polylysine appeared unable to retract. Upon closer examination by thin-section microscopy, we observed that these Tfp structures consisted of multiple aggregates of 8–10 Tfp bundles (Figure 3A–3C). The high density and ordered structure of these bundles enabled us to easily quantify the number of Tfp within a bundle. In bacteria incubated in DMEM alone, the Tfp bundles appeared less ordered (Figures 2C and 3D), and we estimated that each bundle contained up to 8–10 Tfp. Thus, 8–10 Tfp filaments can bundle together to form high force–generating structures (up to forces 8–10 times the force of a single filament retraction force). In this regard, it is interesting to note that N. meningitidis Tfp bundles, some containing 8–10 filaments, are hypothesized to promote bacteria–host cells interactions [21–23].

### Model for the Formation of the Bundles

**Figure 4:**
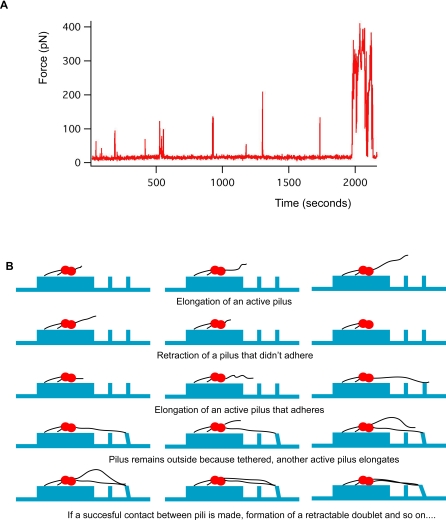
Model for Tfp Bundling and Retraction (A) Progressive rise of force applied by a bacterium on one pillar over time (more than 30 min total time). (B) Cartoon showing the successive association (bundling) of Tfp filaments with each other and their retraction. Timeline begins with top left panel and is read from left to right, top to bottom.

The pattern of force generation suggests that Tfp bundling occurred after an initial anchoring of a single Tfp filament to a surface. The fact that Tfp retraction forces generally increased over time implies that Tfp bundling increased over time as well. Short-term (up to a minute), stepwise increases in the pulling forces (Figure S2A, S2B, and S2D) are indicative of the multiple filament nature of the bundles but do not capture the long-term (∼hours) increase in force generation. Upon careful long-term observation, we detected build-up in pulling force at early attachment sites over extended time (Figure 4A), suggesting that Tfp bundling occurred successively over that time. This did not occur at sites adjacent to these early attachment sites. The early, low-force retractions were transitory, and subsequent higher force retractions always occurred on those initial contact sites rather than on adjacent pillars in the few instances where we could measure the entire force history of a high-force pulling event. This shows that N. gonorrhoeae are able to build strong and lasting contacts on early adhesion sites.

The sum of our observations supports the following model for the generation of high forces through the formation of Tfp bundles (Figure 4B). When a Tfp filament undergoes a cycle of extension and retraction, there are two likely outcomes: either the retraction encounters resistance from the substrate or not. When a single Tfp adheres to a substrate and is not fully retracted, it remains extended. When a second Tfp associates with this extended Tfp (the force of retraction possibly bringing them close to one another), the two can form a retractable doublet that can generate twice the force of a single retractable Tfp. If a third Tfp contacts the doublet, a working triplet is formed. This cooperative mechanism proceeds until a bundle of 8–10 Tfp is formed.

### Conclusion

Using a new micropillar-based assay to measure force, we found that N. gonorrhoeae Tfp filaments form higher-order structures that retract with forces up to 8–10 times higher than previously measured for a single Tfp filament. That a Tfp bundle retracts indicates that retraction of multiple Tfp filaments is coordinated. These studies raise important questions concerning the biochemical interactions among Tfp retraction motors that allow such cooperation [24], as well as the factors that limit bundle size. Our study lends strong support to the notion that the Tfp retraction motor, PilT, is the strongest biological motor known to date. These very strong motors apparently use cooperation as well as relative irreversibility [25] to generate such forces. Those high forces generated by the Tfp can redefine the role of mechanical forces in bacteria–host interactions and perhaps also bacteria–bacteria interactions. The cooperative nature of the mechanisms at play in N. gonorrhoeae Tfp constitutes a new paradigm for the generation of high forces in the biological realm.

## Materials and Methods

### Bacterial strains.

N. gonorrhoeae strains MS11 and MS11*pilT* [26] were used for all experiments and were maintained on GCB agar plus Kellogg's supplements at 37 °C and 5% CO_2_ and passed daily. Piliation and Opa phenotypes were monitored microscopically by assessing colony morphology. Only piliated, Opa–nonexpressing bacteria were used.

### Force measurement devices and analysis.

The pillars were molded from microfabricated wafers containing evenly spaced holes [17]. To ease the demolding, the wafers were silanized with tridecafluoro-trichlorosilane in vapor phase and then plasma cleaned. A droplet of polyacrylamide mix was then sandwiched between an activated coverslip that retained the gel [16] and a piece of wafer bearing the microfabricated holes. After 15 min of curing, the gel was detached from the wafer in 50 mM HEPES. Polyacrylamide to bis-acrylamide concentrations of 20% acrylamide to 0.2% bis and of 20% acrylamide to 1% bis, with the wafers used for this study, resulted, respectively, in pillars with a stiffness constant of 100 ± 30 pN/μm and 500 ± 100 pN/μm, as measured with optical and magnetic tweezers. The analysis of the dynamic motion of the top of the pillars was performed by a home-written correlation plug-in for ImageJ (NIH) with a resolution of 0.3 pixel. The analysis of the static images was performed using the Manual Tracking plugin (Fabrice Cordelières, Institut Curie) with a precision of 1 pixel. The surface of the gel was coated with poly-L-lysine (30 μg/ml, Sigma) using the bifunctional chemical compound sulfo-SANPAH (Pierce) to enable the Tfp to stick to the pillars.

### Sample preparation.

Bacteria were harvested from 14–16-h old agar plates and dispersed in GCB liquid medium at a concentration of 5 × 10^8^ bacteria/ml. A 100-μl volume of that dispersion was added to a 35-mm well of a six-well plate containing a polylysine-coated coverslip or a coverslip with the pillars containing 2 ml of tissue culture medium (DMEM, DMEM + 1mg/ml of BSA or DMEM + 30 μg/ml of poly-L-lysine). The samples were incubated for 3 h at 37 °C, 5% CO_2_, then processed for electron microscopy, fluorescence microscopy, and/or static analysis of Tfp retraction. To analyze the dynamics of Tfp retraction, 1 to 2 μl of the bacteria dispersion was added to 260 μl of relevant medium and sealed between two coverslips spaced approximately 0.5 mm apart. Subsequently, movies were taken at video rate (30 Hz) on an inverted microscope whose temperature was maintained at 37 °C. All experiments were performed at pH 7.4.

### Optical microscopy.

All optical microscope images were obtained on conventional inverted microscopes (either Olympus IX 71 or Zeiss Axiovert 100). Samples processed for fluorescent microscopy were fixed with 3.7% formaldehyde in phosphate-buffered saline (PBS), blocked with a solution of 0.2% fish gelatin in PBS, and incubated with monoclonal antibody 10H5.1.1 that recognizes the conserved (SMI) domain of pilin [27]. All samples were stained with an Alexa-488–conjugated secondary anti-mouse antibody.

### Electron microscopy.

Samples for SEM were fixed with 3.7% formaldehyde in PBS for 1 h, then dehydrated with successive baths in 50%–100% ethanol. They were critical point dried and coated with gold-palladium. Samples for thin-section EM were fixed with 2% glutaraldehyde in PBS for 1 h. They were sequentially exposed to osmium tetroxyde (1%), tannic acid (1%), uranyl acetate (1%), dehydrated with successive baths in 50%–100% ethanol, and finally embedded in an embedding resin. Areas of interest were then glued on a chuck and cut with a microtome.

## Supporting Information

Figure S1 The Two Ways Used for Measuring Retraction Forces (A) Dynamic studies, (B) static studies. Note that the center-to-center distance of the pillars is 3 μm.
(1.71 MB JPG)

Figure S2 Examples of Quantification in the Retraction Process (A–D) Time course of the motion of a pillar showing pauses or successive pulls with discrete forces.
(1.48 MB JPG)

Video S1 Typical N. gonorrhoeae Microcolony Interacting with the Force-Sensing Device This video shows a typical N. gonorrhoeae microcolony interacting with the tips of our micropillar force sensing device. The video is speeded 10 times (×10) and the pilars are 3 μm apart center to center.
(1.8 MB MOV)

Video S2 WT MS11 Incubated in DMEM Only The video is a representative movie of baceria incubated in one specific medium for 3 h on top of an array of pilars of stiffness 500 pN/lm. The video is real time and shows the behavior of one or two bacteria for 23 s after 3 h of incubation. The pilars are 3 lm apart center to center. The video is DIC microscopy with the focus on the top of the pilars. See the already deflected pillars around the bacterium and both transient and sustained pulls.
(5.01 MB MPG)

Video S3 WT MS11 Incubated with BSA The video is a representative movie of baceria incubated in one specific medium for 3 h on top of an array of pilars of stiffness 500 pN/lm. The video is real time and shows the behavior of one or two bacteria for 23 s after 3 h of incubation. The pilars are 3 lm apart center to center. The video is DIC microscopy with the focus on the top of the pilars. See the transient pulls and the fact that the two bacteria are not as tightly connected to the pillars.
(5.5 MB MOV)

Video S4 WT MS11 Incubated with Polylysine The video is a representative movie of baceria incubated in one specific medium for 3 h on top of an array of pilars of stiffness 500 pN/lm. The video is real time and shows the behavior of one or two bacteria for 23 s after 3 h of incubation. The pilars are 3 lm apart center to center. The video is DIC microscopy with the focus on the top of the pilars. See the absence of motions.
(6.12 MB MOV)

## Abbreviations

BSA: bovine serum albumin
DMEM: Dulbecco's modified Eagle's medium
EM: electron microscopy
nN: nanonewton
PBS: phosphate buffered saline
pN: piconewton
SEM: scanning electron microscopy
Tfp: type IV pili
WT: wild type
